# (−)-Epicatechin Prevents Blood Pressure Increase and Reduces Locomotor Hyperactivity in Young Spontaneously Hypertensive Rats

**DOI:** 10.1155/2016/6949020

**Published:** 2016-11-03

**Authors:** M. Kluknavsky, P. Balis, A. Puzserova, J. Radosinska, A. Berenyiova, M. Drobna, S. Lukac, J. Muchova, I. Bernatova

**Affiliations:** ^1^Institute of Normal and Pathological Physiology, Centre of Excellence for Examination of Regulatory Role of Nitric Oxide in Civilization Diseases, Slovak Academy of Sciences, Bratislava, Slovakia; ^2^Institute of Physiology, Faculty of Medicine, Comenius University, Bratislava, Slovakia; ^3^Institute for Heart Research, Slovak Academy of Sciences, Bratislava, Slovakia; ^4^Institute of Medical Physics, Biophysics, Informatics and Telemedicine, Faculty of Medicine, Comenius University, Bratislava, Slovakia; ^5^Institute of Medical Chemistry, Biochemistry and Clinical Biochemistry, Faculty of Medicine, Comenius University, Bratislava, Slovakia

## Abstract

This study investigated the effects of subchronic (−)-epicatechin (Epi) treatment on locomotor activity and hypertension development in young spontaneously hypertensive rats (SHR). Epi was administered in drinking water (100 mg/kg/day) for 2 weeks. Epi significantly prevented the development of hypertension (138 ± 2 versus 169 ± 5 mmHg, *p* < 0.001) and reduced total distance traveled in the open-field test (22 ± 2 versus 35 ± 4 m, *p* < 0.01). In blood, Epi significantly enhanced erythrocyte deformability, increased total antioxidant capacity, and decreased nitrotyrosine concentration. In the aorta, Epi significantly increased nitric oxide (NO) synthase (NOS) activity and elevated the NO-dependent vasorelaxation. In the left heart ventricle, Epi increased NOS activity without altering gene expressions of nNOS, iNOS, and eNOS. Moreover, Epi reduced superoxide production in the left heart ventricle and the aorta. In the brain, Epi increased nNOS gene expression (in the brainstem and cerebellum) and eNOS expression (in the cerebellum) but had no effect on overall NOS activity. In conclusion, Epi prevented the development of hypertension and reduced locomotor hyperactivity in young SHR. These effects resulted from improved cardiovascular NO bioavailability concurrently with increased erythrocyte deformability, without changes in NO production in the brain.

## 1. Introduction

Arterial hypertension is a frequent health problem worldwide. Primary hypertension is detectable in children and adolescents and is increasing in prevalence [1]. Attention deficit hyperactivity disorder (ADHD) is one of the most common developmental disorders that affects approximately 5–7% of children and adolescents [2]. The rate of learning disabilities, including ADHD, is significantly higher for children with sustained primary hypertension as compared to children without hypertension [3]. Arterial hypertension, in addition to other mechanisms, was associated with reduced deformability of erythrocytes, which may participate in the development of both arterial hypertension [4] and behavioral changes.

Spontaneously hypertensive rats (SHR) are a commonly used model of human essential hypertension. SHR also serve as an experimental model of ADHD due to their locomotor hyperactivity and reduced anxiety [5, 6]. However, it is not clear whether the pathways involved in the development of hypertension overlap with those involved in the modulation of locomotor activity. Notably, NO serves as vasodilator in the cardiovascular system (CVS) and as neurotransmitter and neuromodulator in the central and peripheral nervous systems. Therefore, alterations in NO production affect both blood pressure (BP) and behavior. Indeed, neuronal nitric oxide synthase (nNOS) was shown to be involved in various behavioral abnormalities, including ADHD [7, 8].

Oxidative stress is the excessive formation of reactive oxygen species (ROS), especially superoxide (O_2_^∙−^), to a level exceeding the maximal capacity of the antioxidant defense mechanisms of the organism. Oxidative stress has been found to be involved in many disease states, including hypertension and behavioral/mental disorders, in both rodents and humans [9–11]. Superoxide is produced by various physiological aerobic metabolic processes as well as by several enzymatic pathways. One of the main sources of O_2_^∙−^ in living organisms is nicotinamide adenine dinucleotide phosphate oxidase (NADPH oxidase); however, uncoupled nitric oxide synthase (NOS) may also be a significant source of ROS [9]. Furthermore, increased O_2_^∙−^ production may lead to formation of peroxynitrite, a strong prooxidant, which causes peroxynitrite-related cellular damage [12], observed in various cardiovascular disorders [13, 14].

Over the past two decades, there has been increasing interest in the potential health benefits associated with the consumption of flavanol-containing foods [15, 16]. Several studies have reported that the consumption of flavanol-containing foods was associated with a lower prevalence of cardiovascular diseases [17, 18]. Furthermore, several meta-analyses have confirmed a BP-lowering capacity and antihypertensive effect of flavanol-rich foods derived from cocoa [19, 20]. BP-lowering effect of cocoa-derived products depended on the dose of ingested (−)-epicatechin (Epi) [21].

Epi is absorbed well from the gastrointestinal tract in both humans and rats, detectable in plasma approximately 30 min after ingestion. Epi concentrations peak 2-3 h after ingestion and return to baseline by 6–8 h [22, 23], suggesting that the continuous intake of Epi-containing food is needed to maintain elevated circulation levels. The BP-reducing effect of Epi was shown in models of L-NAME-induced [24], fructose-induced [25], and DOCA-salt hypertension [26]. We have previously observed that Epi reduces BP and improves endothelium-dependent vasorelaxation in adult SHR with fully developed hypertension via improved vascular NO bioavailability [27].

Regarding the role of Epi in the central nervous system (CNS), recent studies have demonstrated that Epi can cross the blood-brain barrier (BBB) and enter the brain [28, 29], which may result in altered CNS function. Studies have also shown that prolonged cocoa flavanol consumption improves cognitive function, blood pressure control, and metabolic profile in elderly subjects [30]. Although the underlying mechanism responsible for the observed effects of cocoa-derived foods on the CNS remains unknown, it may be associated with improved NO bioavailability, vascular function, and/or increased erythrocyte deformability, which all together may improve organ perfusion.

Therefore, the aim of this study was to investigate whether the subchronic treatment of peripubertal SHR with Epi may prevent the development of hypertension and locomotor hyperactivity in this genetic model of hypertension and ADHD. To elucidate the mechanism(s) of Epi action, we investigated superoxide and NO production as well as the gene expression of the p22phox subunit of NADPH oxidase and individual NOS isoforms in the CVS and selected regions of the brain, total antioxidant capacity of plasma, nitrosative damage, the deformability of erythrocytes, and vascular function.

## 2. Material and Methods

### 2.1. Animals and Treatment

Young 5-week-old SHR males (*n* = 18) were used. All rats were born in our certified animal facility (Institute of Normal and Pathological Physiology SAS) in order to maintain the same environmental background for all animals. The rats were housed two per cage at constant temperature 22–24°C and humidity (45–60%) with a 12 : 12 h light-dark cycle (lights on from 06.00 a.m. to 06.00 p.m.) and fed a standard pellet diet with tap water* ad libitum*. At the beginning of the experiment (Basal, B), rats were randomly assigned to the control group (Cont, *n* = 8) or a group treated with Epi (Epi, *n* = 10). Epi was administered to rats diluted in the appropriate daily volume of water, in concentration that resulted in a final daily dose of Epi approximately 100 mg/kg body weight/day, for two weeks. Daily volume of water was assessed for each cage of rats prior to starting the experiment and adjusted daily. Average daily drinking volume of rats was 17 ± 0.8  mL/100 g of body weight and Epi did not influence it. Concentrated Epi solution (100 mg/mL) was prepared fresh every day before administration to rats by dilution of Epi in tap water (85°C, 3 min, in water bath). Calculated volume of concentrated Epi solution was added to assessed volume of fresh tap water in the bottles of rats to reach the dose 100 mg/kg body weight/day after drinking out all liquid during 24 h period. Concentration of Epi in bottles was approximately 0.58 mg/mL. If rats drank the given volume of liquid earlier, fresh water was added to the bottle to prevent thirst and/or stress from the lack of water. Epi solutions (both concentrated and diluted in bottles) were protected against the light. Fresh Epi was administered to rats at the end of the light period, as the majority of drinking activity of rodents occurs in the dark (active) period [31]; thus approximately 80% of solution was drunk during the dark period and the rest in the light period. Thermal and time-dependent stability of Epi in water has been shown previously [32].

At the end of the 2-week treatment, the rats were exposed to brief CO_2_ anesthesia. Rats were subsequently killed by decapitation, and trunk blood was collected to evaluate erythrocyte deformability, nitrotyrosine concentration, and total antioxidant capacity. Wet mass of the left heart ventricle (LHV) was determined to calculate relative weight (LHV/body weight) in order to ascertain the degree of LHV hypertrophy.

All procedures were performed in accordance with the institutional guidelines and approved by the Department of Animal Wellness, State Veterinary and Food Administration of the Slovak Republic.

### 2.2. Blood Pressure and Heart Rate

Systolic blood pressure and heart rate (HR) were measured in preconditioned, conscious rats by noninvasive tail-cuff plethysmography between 08:00 a.m. and 11:00 a.m. as described in detail previously [33]. Each value was calculated as the average of five measurements. BP values were measured repeatedly at the beginning of the experiment (B) and after the seventh, tenth, and fourteenth day of treatment. Body weight (BW) was determined on the same days.

### 2.3. Open-Field Test

Rat motor activity and anxiety level were measured using the open-field test (OF) between 07:30 a.m. and 10:00 a.m. The open-field apparatus comprised a 100 × 100 cm area with a black floor and black walls (50 cm high) with a virtual central zone (55 × 55 cm) and corners (12.5 × 12.5 cm). The OF was illuminated by warmwhite light at 150 lx. Rats were placed in the centre of the OF; motor activity was recorded and evaluated by ANY-maze video-tracking software (Stoelting, USA) during 10 min trials. The OF area was cleaned with soapy water and dried with paper towels after each trial. The following behavioral parameters were determined: total distance traveled, total time of immobility, central zone distance traveled, and time spent in the central zone and in the corners. Average speed was calculated as the ratio of total distance traveled to time of mobility for a given rat. As anxiety markers, relative central zone distance (calculated as the percentage of central zone distance with respect to total distance traveled) and relative central zone time (calculated as the percentage of time spent in the central zone with respect to total mobility time) were determined [34].

All rats were tested one day before the beginning of the experiment to determine baseline measurements (B) in 5-week-old rats. Rats were randomly assigned to the control or Epi-treated group and tested again two days before the end of the experiment (~7 weeks of age). Thus, OF behavior was determined one day prior to BP measurement to avoid the effect of the OF test on BP level. All cages with rats were placed into a test room with the lighting and environmental conditions described above, approximately 12 h before the test.

### 2.4. Erythrocyte Deformability, Total Antioxidant Capacity of Plasma, and Nitrotyrosine Concentration

Trunk blood samples were collected in heparinized test tubes and immediately thereafter centrifuged at 850 ×g for 10 min at 4°C to obtain plasma and erythrocytes. Plasma was separated, aliquoted, and stored at −80°C until the time of analysis.

After removing the plasma, the buffy coat and upper 20% of packed red blood cells were removed by aspiration. The remaining erythrocytes were washed three times in manufacturer-formulated Cellpack solution (diluent for Sysmex blood analyser, Sysmex F–820, Japan). The washed erythrocytes were diluted in Cellpack solution (1 : 1, v : v) and adjusted to 30–40% hematocrit. The diluted suspension of erythrocytes was filtered by centrifugation through membrane filters with pores of 5 *μ*m in diameter (Ultra-free-MC SV Centrifugal Filter, Millipore, Germany) at 1400 rpm (Hettich MIKRO 120 centrifuge). Erythrocyte deformability was calculated as the percentage of filtered erythrocytes with respect to the number of erythrocytes counted before centrifugation [35].

The total antioxidant capacity (TAC) of plasma was measured by determining the trolox equivalent antioxidant capacity as described previously by [36]. Quantification was performed using the dose-response curve for the reference antioxidant trolox, which is a water-soluble form of vitamin E. The results are presented as mmol of trolox/L. TAC was determined in six control and six Epi-treated rats.

Concentration of nitrotyrosine in plasma was detected by ELISA using commercially available kit (HK501-02, Hycult Biotech, Uden, Netherlands) according to the manufacturer's protocol. Absorbance of the plasma samples was measured at 450 nm. The nitrotyrosine concentration of samples was determined from the standard curve and expressed in nmol/L.

### 2.5. Superoxide Production

The production of superoxide (O_2_^∙−^) was measured in tissue samples of the LHV and thoracic aorta (15–20 mg) by lucigenin-enhanced chemiluminescence (50 *μ*mol/L) using a TriCarb 2910TR liquid scintillation analyser (Perkin Elmer), as described previously [37]. The results are expressed as counts per minute per milligram of tissue (cpm/mg).

### 2.6. Nitric Oxide Synthase Activity

Total NOS activity was measured in the 20% tissue homogenates of the LHV, aorta, brainstem, and cerebellum by determining [^3^H]-L-citrulline formation from [^3^H]-L-arginine (MP Biomedicals, USA) as described previously [33] and expressed as pmol/min/mg of tissue proteins as determined using the Lowry method. NOS activity was determined in six control and eight Epi-treated rats.

### 2.7. Vascular Function

The vascular reactivity of the aorta was investigated as described previously [38]. Endothelium-dependent vasorelaxant responses were examined in rings precontracted with phenylephrine (3 *μ*mol/L) to produce a stable plateau of contraction. After a contraction plateau had been reached, increasing concentrations of acetylcholine (ACh, 0.001–10 *μ*mol/L) were added cumulatively. When the ACh-induced concentration-relaxation curve was completed, the drugs were washed out (20 min), and the same experiment was repeated after 25 min preincubation with NO synthase inhibitor N^G^-nitro-L-arginine methyl ester (L-NAME, 300 *μ*mol/L). After this procedure and a 30 min washout period, the NO donor sodium nitroprusside (SNP, 0.001–10 *μ*mol/L) was added cumulatively to the 3 *μ*mol/L phenylephrine precontracted aortae. The extent of vasorelaxation was expressed as the percentage change with respect to stable phenylephrine-induced contraction.

NO-independent component of endothelium-dependent ACh-induced relaxation was determined as the rest of relaxation present after inhibition of vascular NO production with L-NAME and expressed as the area under the concentration-response curve (AUC), in arbitrary units (a.u.). Endothelium-dependent ACh-induced relaxation mediated by NO (i.e., NO-dependent component) was calculated as the difference in the AUC before L-NAME pretreatment (i.e., total ACh-induced relaxation) and after L-NAME pretreatment (i.e., NO-independent relaxation). AUC was calculated from individual concentration-response curves, as it was described in detail previously [33].

### 2.8. Gene Expression

Expression levels of neuronal NOS (nNOS), inducible NOS (iNOS), and endothelial NOS (eNOS) as well as p22phox (a transmembrane subunit of NADPH oxidase) were investigated by real-time quantitative polymerase chain reaction (RT-qPCR) using a CFX96 Real-Time PCR detection system (Bio–Rad, USA). Total RNA from the brainstem, cerebellum, and LHV samples was isolated using TRIsure reagent (Bioline, United Kingdom) according to the manufacturer's protocol. The amount of total RNA isolated was quantified spectrophotometrically at 260/280 nm using a NanoDrop spectrophotometer (Thermo Scientific, USA).

For reverse transcription (Eppendorf Mastercycler, Germany), 1 *μ*g of total RNA was added to 20 *μ*L of reaction medium using a SensiFAST™ cDNA Synthesis Kit (Bioline, UK) according to the manufacturer's protocol.

The primer pair specifications used to amplify the genes studied (nNOS, iNOS, eNOS, and p22phox, resp.) as well as a housekeeping gene (*β*-actin) are listed in Table 1. The PCR mixture contained 1.5 *μ*L of template cDNA diluted tenfold, 10 *μ*L SensiFAST mix (SensiFAST SYBR No–ROX kit, Bioline, UK), 1.5 *μ*L of both forward and reverse primers (Metabion, Germany, 4 *μ*mol/L), and 5.5 *μ*L diethylpyrocarbonate-treated water (Sigma–Aldrich, Germany) in a final volume of 20 *μ*L. The thermal cycling conditions were as follows: (1) 50°C for 2 min, (2) 95°C for 2 min, (3) 39 cycles consisting of (a) 95°C for 5 sec, (b) an optimal annealing temperature (depending on the selected primer, see Table 1) for 10 sec, and (c) 72°C for 5 sec for PCR product elongation, and (4) 72°C for 1.5 min. Finally, melt curves for amplicon analyses were constructed at 50–99°C, 10 sec/1°C. Samples were measured using Bio–Rad CFX Manager software (version 2.0) and *β*-actin as the housekeeping gene. Gene expression was determined in six control and eight Epi-treated rats and expressed as the ratio of gene expression with respect to *β*-actin levels.

All chemicals used in this study were purchased from Sigma–Aldrich (Germany) and Merck Chemicals (Germany), if not stated differently. Epi was purchased from Sigma (Germany, Cat. no. E1753).

### 2.9. Statistical Analysis

Results were analysed by unpaired Student* t*-test or one-way analysis of variance (ANOVA) where appropriate. BP, HR, and BW were analysed by two-way ANOVA (treatment × time). Vascular function was analysed by two-way ANOVA (treatment × ACh concentration). All ANOVA analyses were followed by the Bonferroni* post hoc* test. Values were considered to differ significantly when *p* < 0.05. Data are presented as mean ± standard error of the mean (SEM). Correlations between variables were determined using Pearson's correlation coefficient (*r*). GraphPad Prism 5.0 (GraphPad Software, Inc., USA) and Statistica 7 (Stat Soft, Inc., USA) were used for the statistical analyses.

## 3. Results

**Figure 1:**
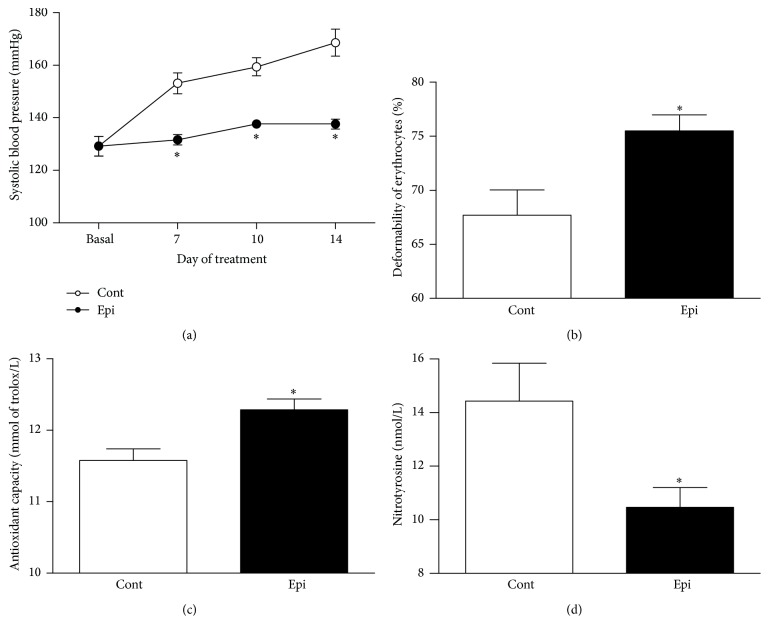
Effect of (−)-epicatechin on systolic blood pressure (a), deformability of erythrocytes (b), total antioxidant capacity of plasma (c), and plasma nitrotyrosine concentration (d) in spontaneously hypertensive rats. ^*∗*^ *p* < 0.05 versus Cont group. Values represent mean ± SEM; *n* = 6–8 for Cont and *n* = 6–10 for Epi. Abbreviations: Cont: control group and Epi: (−)-epicatechin-treated group.

Two-week Epi treatment had no effect on the increase in BW controlled for age (data not shown). Relative weight of the LHV was similar in the Epi (2.21 ± 0.07 mg/g) and control (2.31 ± 0.06 mg/g) groups. BP was reduced by approximately 18% in Epi-treated rats as compared to controls at the end of treatment (Figure 1(a)). Epi treatment reduced heart rate only on the 10th day of treatment (571.8 ± 17 bpm in control versus 524.8 ± 8.2 bpm in Epi, *p* < 0.05) while only nonsignificant difference (540 ± 19 bpm versus 530 ± 9 bpm) was observed on day 14. In addition, Epi increased erythrocyte deformability by approximately 8% (*p* < 0.05), increased the TAC (*p* < 0.05), and reduced nitrotyrosine concentration in plasma (*p* < 0.05) versus controls (Figures 1(b), 1(c), and 1(d)).

**Figure 2:**
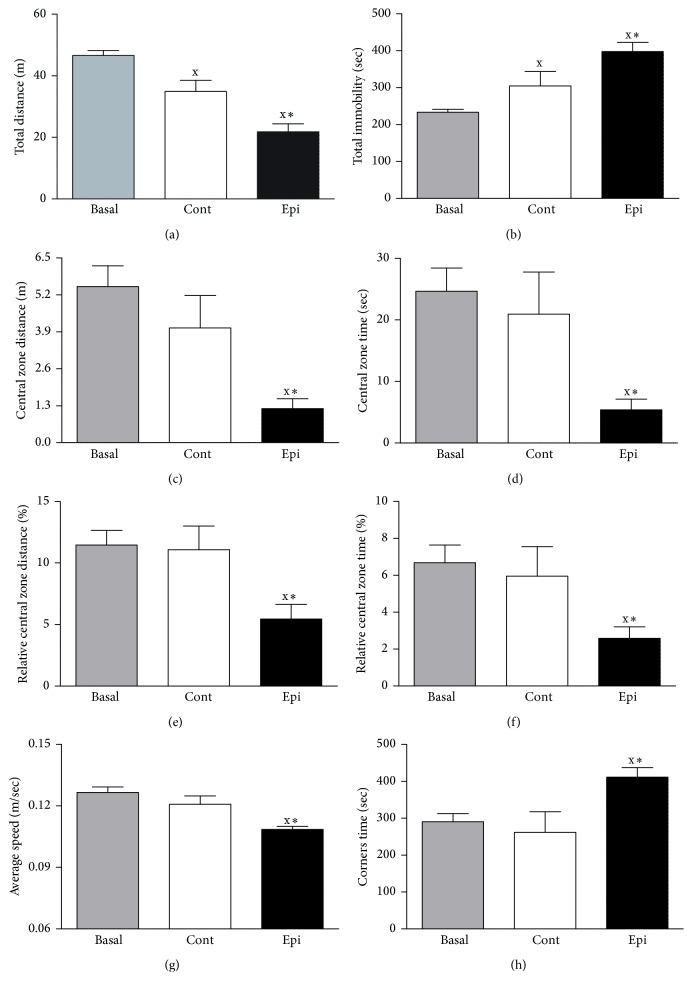
Effect of (−)-epicatechin treatment on open-field behavior of spontaneously hypertensive rats. Total distance traveled (a), total immobility (b), distance traveled in the central zone (c), time spent in the central zone (d), relative central zone distance (e), relative central zone time (f), average speed (g), and time spent in the corners (h). Values represent mean ± SEM; *n* = 18 for Basal, *n* = 8 for Cont, and *n* = 10 for Epi. ^x^ *p* < 0.05 versus Basal values; ^*∗*^ *p* < 0.05 versus Cont group. Abbreviations: Cont: control group and Epi: (−)-epicatechin-treated group.

Regarding rat behavior, repeated testing in the open-field at the end of experiment led to habituation of locomotor activity detected as reduction of total distance traveled and increase of total immobility compared to Basal values (Figures 2(a) and 2(b)). Epi administration led to a significant decrease in locomotor activity as represented by total distance traveled (Figure 2(a)), increased immobility (Figure 2(b)), and reduced the average speed of movement (Figure 2(g)) in treated animals as compared to age-matched controls. Epi decreased the distance traveled (Figure 2(c)) and time spent in the central zone (Figure 2(d)). In addition, Epi reduced both relative distance traveled and relative time spent in the central zone (Figures 2(e) and 2(f)). Epi treatment also significantly elevated time spent in the corners (Figure 2(h)) in treated animals as compared to controls.

**Figure 3:**
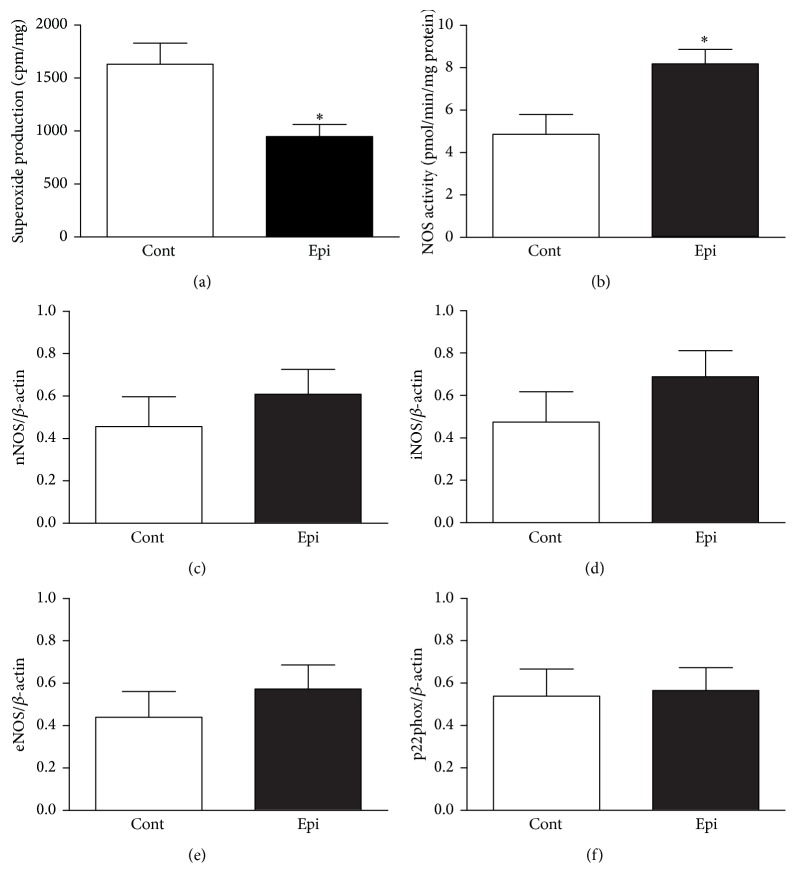
Effect of (−)-epicatechin treatment on superoxide production (a), nitric oxide synthase (NOS) activity (b), gene expression of neuronal NOS (nNOS, c), inducible NOS (iNOS, d), endothelial NOS (eNOS, e), and the p22phox subunit of nicotinamide adenine dinucleotide phosphate oxidase (f) in the left heart ventricle of spontaneously hypertensive rats. Values represent mean ± SEM; *n* = 6–8 for Cont and *n* = 8–10 for Epi. ^*∗*^ *p* < 0.05 versus Cont group. Abbreviations: Cont: control group and Epi: (−)-epicatechin-treated group.

Epi significantly reduced O_2_^∙−^ production and increased NOS activity in the LHV (Figures 3(a) and 3(b)). However, Epi failed to affect gene expression levels for individual NOS isoforms (Figures 3(c)–3(e)) and p22phox (Figure 3(f)) in the LHV.

**Figure 4:**
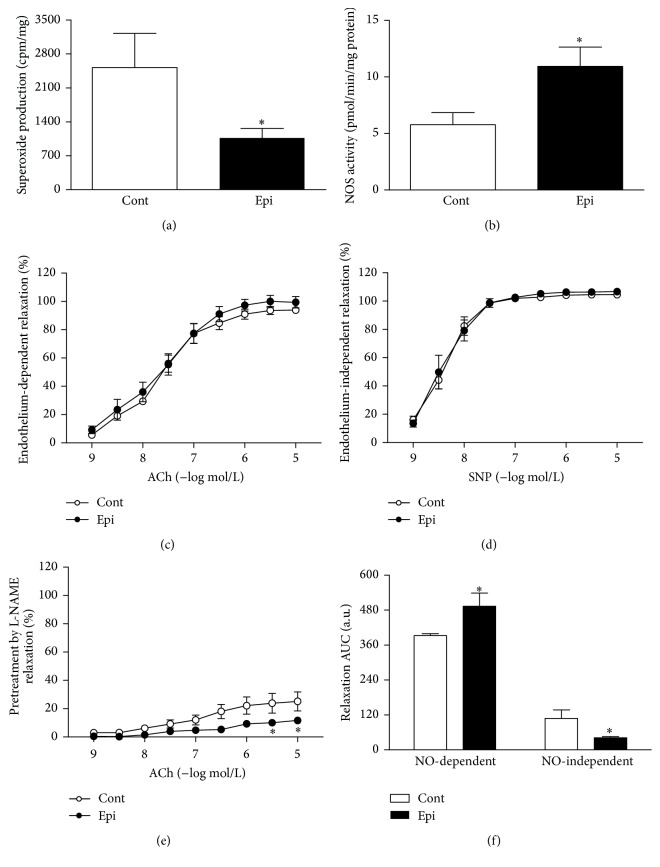
Effect of (−)-epicatechin treatment on superoxide production (a), NOS activity (b), endothelium-dependent relaxation induced by acetylcholine (c), endothelium-independent relaxation induced by SNP (d), inhibitory effect of L-NAME pretreatment (300 *μ*mol/L) on ACh-induced relaxation (e), and NO-dependent and NO-independent components of relaxation (f) in the aorta of spontaneously hypertensive rats. Values represent mean ± SEM; *n* = 6–8 for Cont and *n* = 6–10 for Epi. ^*∗*^ *p* < 0.05 versus Cont group. Abbreviations: ACh: acetylcholine, AUC: area under the curve, a.u.: arbitrary units, Cont: control group, Epi: (−)-epicatechin-treated group, L-NAME: N^G^-nitro-L-arginine methyl ester, NO: nitric oxide, NOS: nitric oxide synthase, and SNP: sodium nitroprusside.

In the aorta, Epi significantly reduced O_2_^∙−^ production and increased NOS activity (Figures 4(a) and 4(b)). Neither endothelium-independent relaxation responses induced by SNP nor overall endothelium-dependent relaxation induced by ACh differed significantly in the aortae of control as compared to Epi-treated rats (Figures 4(c) and 4(d)). Acute L-NAME pretreatment, which inhibited NO-dependent relaxation, inhibited relaxation more strongly in Epi-treated rats as compared to controls (Figure 4(e)). Calculation of the AUC revealed that Epi significantly increased endothelial NO-dependent relaxation by approximately 26% versus control and concurrently decreased endothelial NO-independent relaxation in the aorta (Figure 4(f)).

**Figure 5:**
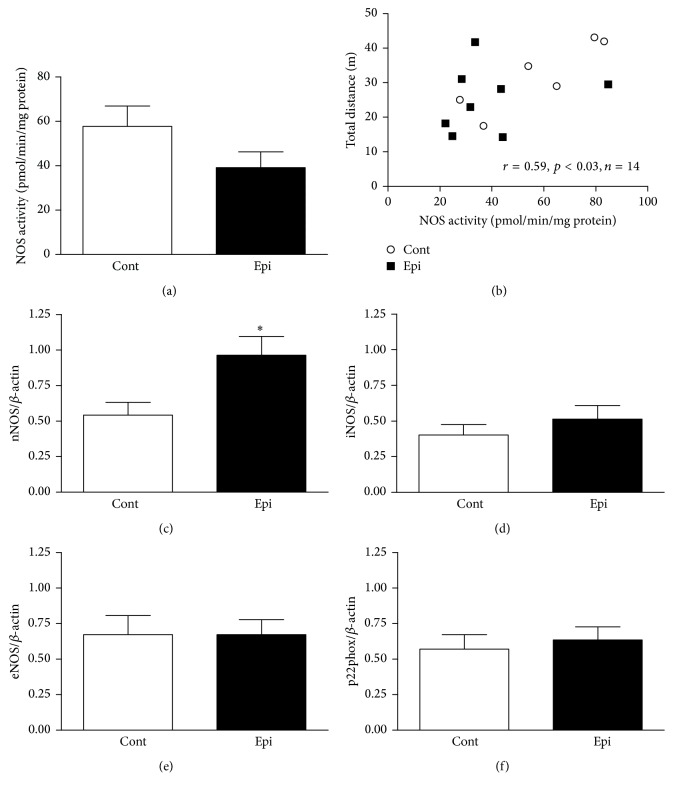
Effect of (−)-epicatechin treatment on nitric oxide synthase activity (a), correlation between total distance traveled in the open-field and nitric oxide synthase activity (b) and gene expression of nNOS (c), iNOS (d), eNOS (e), and the p22phox (f) in the brainstem of spontaneously hypertensive rats. Values represent mean ± SEM; *n* = 6 for Cont and *n* = 8–10 for Epi. ^*∗*^ *p* < 0.05 versus Cont group. Abbreviations: Cont: control group, Epi: (−)-epicatechin-treated group, NOS: nitric oxide synthase, eNOS: endothelial NOS, iNOS: inducible NOS, nNOS: neuronal NOS, and p22phox: subunit of nicotinamide adenine dinucleotide phosphate oxidase.

**Figure 6:**
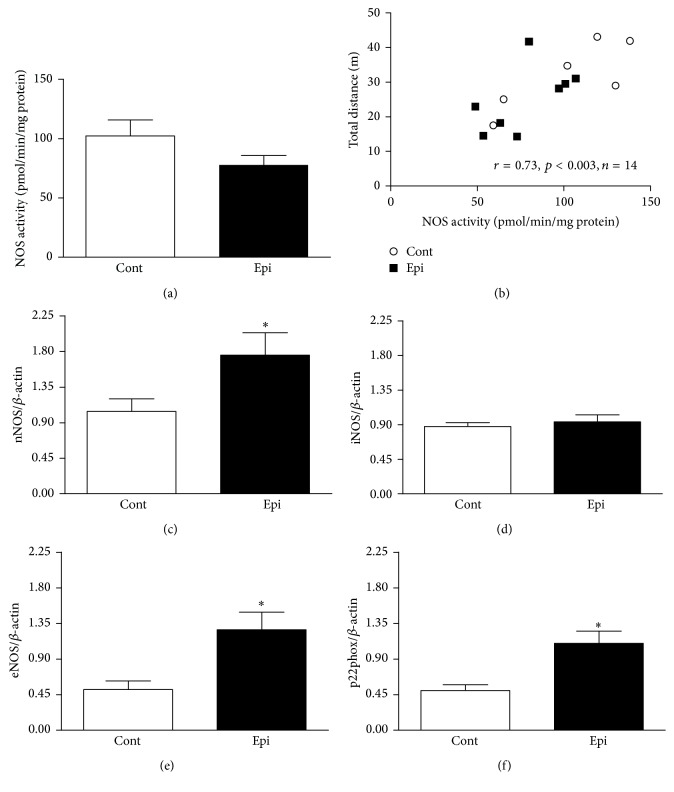
Effect of (−)-epicatechin treatment on nitric oxide synthase (NOS) activity (a), correlation between total distance traveled in the open-field and NOS activity (b) and gene expression of nNOS (c), iNOS (d), eNOS (e), and p22phox (f) in the cerebellum of spontaneously hypertensive rats. Values represent mean ± SEM; *n* = 6 for Cont and *n* = 8–10 for Epi. ^*∗*^ *p* < 0.05 versus Cont group. Abbreviations: Cont: control group, Epi: (−)-epicatechin-treated group, eNOS: endothelial NOS, iNOS: inducible NOS, nNOS: neuronal NOS, and p22phox: subunit of nicotinamide adenine dinucleotide phosphate oxidase.

NOS activity was unaffected by Epi in both brain regions investigated (brainstem and cerebellum) (Figures 5(a) and 6(a)). Interestingly, NOS activity in the brainstem and cerebellum correlated positively with total distance traveled (as shown in Figures 5(b) and 6(b)) as well as with central zone distance traveled in the OF (*r* = 0.53, *p* < 0.05, *n* = 14 for brainstem; *r* = 0.62, *p* < 0.02, *n* = 14 for cerebellum).

Gene expression levels of nNOS increased (Figures 5(c) and 6(c)) while iNOS levels remained the same in both brain regions investigated in Epi-treated rats (Figures 5(d) and 6(d)). Interestingly, eNOS and p22phox gene expression were increased in the cerebellum of Epi-treated rats as compared with controls (Figures 6(e) and 6(f)). This effect was not observed in the brainstem.

## 4. Discussion

This study investigates the effect of subchronic treatment with Epi in young SHR rats, at the peripubertal age, which is a critical developmental period when BP increases rapidly in SHR [39, 40]. We show that continuous Epi treatment during this period significantly prevented BP increase and reduced spontaneous locomotor hyperactivity in SHR. In addition, we show here for the first time that Epi treatment increased erythrocyte deformability in SHR.

In this study, Epi was administered continuously in tap water. It is known that Epi is subject to partial degradation in water and further metabolism after ingestion. The presence of Epi and/or its metabolites (e.g., 3′-O-methyl epicatechin and 4′-O-methyl epicatechin) in plasma as well as in the brain was detected previously after administration of the same dose of Epi as used in this study [41]. However, despite the fact that biologically active substance(s) may differ from Epi itself, this study demonstrates the significant biological effects of orally administered Epi. We used the given dose of Epi (100 mg/kg/day), as we were interested in possible central effects of Epi, despite the fact that BP-lowering effect can be reached by lower doses. As the relatively high dose of Epi was used in this study, we determined creatinine, uric acid, and urea in plasma at the end of Epi treatment to reveal whether the given dose of Epi is safe or if it produces adverse side effects to kidneys. No signs of renal toxicity of the given dose of Epi were observed in our study (see Supplementary Materials available online at http://dx.doi.org/10.1155/2016/6949020).

The Epi-mediated prevention of hypertension development was associated with elevated plasma TAC and reduced superoxide production in the LHV and aortae of Epi-treated rats. However, these findings were not associated with changes in the gene expression of the p22phox subunit of NADPH oxidase, one of the main sources of O_2_^∙−^ in the CVS. These findings support other studies in which the antioxidant capacity of Epi was associated either with activation of the enzymes involved in the antioxidant defense system [42] in the heart or with radical-scavenging properties in endothelial cells without affecting NADPH oxidase activity* in vitro* [43, 44]. On the other hand, short-term Epi cotreatment reduced protein expression levels of the p47phox subunit of NADPH oxidase in the hearts of rats with L-NAME-induced hypertension [24] and in the renal cortex in fructose-fed rats [45], in contrast to our findings in a genetic model of hypertension. Regarding nitrosative damage,* in vitro* studies revealed that Epi protected cells against peroxynitrite-induced damage [46, 47] similarly as we observed* in vivo* in blood.

In addition to the reduction in superoxide production and increased NOS activity in the aorta and LHV as well as reduced plasma nitrotyrosine concentration, increased aortic endothelial NO-dependent relaxation also proofs better NO bioavailability in the CVS. Interestingly, no effect of Epi on e/i/nNOS gene expression in the LHV was found in this study, suggesting that Epi influences the catalytic properties of NOS but not its gene expression in the CVS. A similar mechanism was demonstrated previously in cultured endothelial cells [48] as well as in the cardiac tissue of L-NAME-treated rats [24]. Therefore, our study in a genetic model of spontaneous hypertension confirms the ability of Epi to increase the CVS capacity for NO production resulting in elevated NO bioavailability; however, the involvement of individual NOS isoforms remains to be clarified.

Regarding vascular function, acute Epi administration induces both endothelium-dependent and endothelium-independent relaxation in the isolated arteries of normotensive rats and in human arteries [49–51]. The Epi-induced endothelium-dependent relaxation in normotensive rats was primarily mediated by NO [49, 50]. Recent study of Moreno-Ulloa et al. has suggested G protein-coupled estrogen receptor (GPER) as a potential mediator of Epi effects in vasculature, which was associated with elevated phosphorylation of eNOS in Wistar rats [52]. We have shown recently that relatively short-term (10-day) dietary administration of Epi reversed endothelial dysfunction in the femoral artery of adult SHR by enhancing the NO-dependent component of relaxation [27]. Similarly, in people with never-treated essential hypertension, administering flavanol-rich dark chocolate (which has a high concentration of Epi) for two weeks normalized the NO-mediated endothelium-dependent relaxation in the brachial artery [53]. In this study, Epi elevated NOS activity in the aorta and enhanced the NO-dependent component of ACh-induced relaxation but failed to affect overall relaxation in peripubertal SHR. Yet despite the lack of an effect on overall relaxation in the aorta, the improvements in vascular NO bioavailability and the NO-dependent component of relaxation observed in Epi-treated rats in this study may prevent vascular remodeling and reduce vascular wall stiffness [54], both of which are observed in hypertension [55–57].

Furthermore, the increased level of erythrocyte deformability observed in this study suggests improvements in blood flow as well as oxygenation in individual organs, similarly as has been recently observed in humans after two-week cocoa flavanol intake [58]. As erythrocytes contain functional eNOS and NO increases their deformability [59], it is plausible that the positive health effects of Epi are associated with the NO-related modulation of red blood cell properties [60]. Collectively, these observations in various experimental conditions both* in vitro* and* in vivo* suggest multiple mechanisms for the cardioprotective effects of Epi that are associated with improved NO bioavailability in the heart and vasculature as well as with enhancement of mechanical properties of the red blood cells.

In addition to the cardiovascular effects of Epi, our study pointed also to possible central effects. As mentioned above, Epi can cross the BBB [28, 29, 41]. Moreover, BBB was shown to be damaged in hypertension, specifically, in the brainstem and cerebellum [56, 61]. We chose these areas of the brain to focus on because the cerebellum integrates the neural control of movement and plays a role in the pathogenesis of ADHD [62]. The brainstem was selected as it is a part of the brain involved in the control of bodily motor function, in addition to the regulation of cardiac and respiratory functions.

In humans, the consumption of natural polyphenols, including cocoa flavanols, results in an acute improvement in visual and cognitive functions [63, 64], which may be relevant in the treatment of ADHD. Indeed, Pycnogenol®, a polyphenol extract from the bark of the French maritime pine, significantly reduces hyperactivity and improves attention, visual-motor coordination, and concentration in children with ADHD [65]. However, to our knowledge, the effect of Epi on ADHD symptoms has not yet been investigated in humans. Several studies in rodents have demonstrated the variable effects of flavanols on behavior. In Wistar rats, a single dose of cacao mass showed anxiolytic effects, but 2-week consumption did not reduce anxiety-related behavior. Locomotor activity in the OF was unaffected in those rats [66]. Two-week cocoa polyphenolic extract treatment had an antidepressant-like effect in Wistar-Unilever rats subjected to a forced-swim test without accompanying changes in locomotion in the OF [67]. In adult C57BL/6 mice, Epi had an anxiolytic effect as represented by an elevated ratio of distance traveled and time spent in the central zone of the OF compared to periphery [68]. However, it has to be noted that all of these studies were performed in normotensive rodents. We used SHR, which are known to be locomotor hyperactive with high levels of exploratory activity and reduced levels of anxiety compared to normotensive rat strains [5, 69, 70]. In our study, Epi administration attenuated locomotor hyperactivity as determined by decreases in the total distance traveled and the average speed of movement as well as by increases in total immobility. Epi also deceptively elevated anxiety in the OF, as suggested by reductions in total distance traveled and time spent in the central zone (in both absolute and relative values) and increased time spent in the corners. However, considering the innate hyperactivity and low anxiety levels of control SHR, Epi, in fact, corrected their behavioral abnormalities. These alterations were not associated with changes in NOS activity in the selected brain areas. However, in contrast to our findings in the LHV, we observed increased nNOS gene expression in both areas of the brain investigated here; eNOS gene expression increased only in the cerebellum. Interestingly, NOS activity in the brainstem and cerebellum correlated positively with locomotor activity and negatively with anxiety level (determined as a reduction in the central zone distance traveled) in the OF. These correlations were stronger in the cerebellum, suggesting that cerebellar NO-dependent mechanisms are more significantly involved in modulation of locomotor activity in young SHR. Yet, the studies performed to date in rats and in humans have demonstrated the considerable variability of findings on the role of NO in the modulation of behavior as well as the varying effects of NO in different neuroanatomical structures of the brain, which might even be antagonistic on the behavioral level [8, 34].

It is of interest that gene expression levels for the p22phox subunit of NADPH oxidase were increased in the cerebellum following Epi treatment, which is in contrast to the findings reported for the CVS in different animal models of hypertension [24–26]. If the Epi-induced upregulation of NADPH oxidase gene expression was to be followed by translation into functional enzyme, the abovementioned antioxidant effects of Epi could still maintain ROS at physiological levels. Our review of the literature did not reveal any study that has investigated the effect of subchronic Epi treatment on e/i/nNOS or p22phox NADPH oxidase subunit gene expression or activity in the CVS or brain of SHR. Yet our findings suggest that Epi exerts tissue-specific effects on the expression of individual NOS isoforms and NADPH oxidase subunits in SHR. These effects may not correlate with enzyme activity levels in the corresponding tissue [71].

The simultaneous prevention of BP increase and reduced hyperactivity of SHR observed in this study suggest the possibility of a common mechanism(s) underlying both pathologies. One possible mechanism is a reduction in noradrenergic neurotransmission, which is elevated in SHR and associated with high blood pressure and locomotor hyperactivity [62, 72]. In Epi-treated SHR rats, noradrenergic hyperfunction may be diminished by presynaptic *α*_2_-autoreceptor-mediated feedback [62, 73], the improvement of calcium signaling [62, 74], and/or increases in bioavailability of NO [75]. These effects may prevent hypertension development and decrease locomotion in young SHR. Another plausible mechanism is the improvement of regional cerebral blood flow, as its alterations were observed in SHR and children with ADHD [62, 76] and flavanol-rich cocoa consumption improved it in older healthy volunteers [77].

Although our study brought interesting results related to simultaneous prevention of hypertension and reduction of behavioral hyperactivity in juvenescent rats, there are certain limitations of this study. Firstly, NO production, gene expressions, and vascular function were determined in the aorta. These parameters may differ in smaller arteries, so the effect of Epi, especially in the small resistance arteries, needs to be investigated. Secondly, we did not determine Epi and/or Epi metabolites levels in blood, NOS phosphorylation, and involvement of GPER receptors. Thus, further studies are needed to elucidate the exact bioactive substance(s) and the exact site(s) of action of orally administered Epi in preventing and treating hypertension and behavioral hyperactivity in young subjects.

## 5. Conclusion

In conclusion, the results presented here showed that oral Epi treatment significantly prevented BP increase and reduced behavioral hyperactivity in young SHR. The mechanism underlying the positive effects of Epi observed in this study was related to improved cardiovascular NO bioavailability, due to elevated NOS activity and reduced O_2_^∙−^ levels in the CVS concurrently with elevations in plasma antioxidant capacity as well as red blood cell deformability. Altogether, these beneficial alterations could result in reduced sympathetic tone and improved cerebrovascular blood flow and tissue oxygenation, resulting in the prevention of hypertension and the reduction of locomotor hyperactivity. The results of this study may be relevant in pharmacological approaches to the prevention and treatment of hypertension and ADHD comorbidity in young subjects with a significant family history of hypertension. Our data also suggest tissue-specific influences of Epi in SHR that should be taken into account in evaluating the overall effects of Epi-containing foods.

## Supplementary Material

Creatinine, uric acid and urea were determined in blood plasma at the end of treatment. All parameters were measured in the accredited medical laboratory Synlab Slovakia Ltd. (Bratislava, Slovakia). Results are expressed as mean ± SEM and they were analyzed by Student's T-test.

## Figures and Tables

**Table 1:** Primer pairs used to amplify selected genes.

Genes	Forward (sense) primer	Reverse (antisense) primer	Temp
eNOS	CCC ACA GTC TGG TTG CT	TCA CCG TGC CCA TGA GT	57°C
iNOS	TGG AGG TGC TGG AAG AGT T	GGA GGA GCT GAT GGA GTA GT	57°C
nNOS	CGC TAC GCG GGC TAC AAG CA	GCA CGT CGA AGC GGC CTC TT	60°C
*β*-actin	AAT CGT GCG TGA CAT CAA AG	ATG CCA CAG GAT TCC ATA CC	57°C
p22phox	CAG GCA TAT ACC CGC TAC CT	TCT GTC ACC CTG TGC TTG AC	60°C
