# Corrigendum to “(−)-Epicatechin Prevents Blood Pressure Increase and Reduces Locomotor Hyperactivity in Young Spontaneously Hypertensive Rats”

**DOI:** 10.1155/2017/9483845

**Published:** 2017-04-20

**Authors:** M. Kluknavsky, P. Balis, A. Puzserova, J. Radosinska, A. Berenyiova, M. Drobna, S. Lukac, J. Muchova, I. Bernatova

**Affiliations:** ^1^Institute of Normal and Pathological Physiology, Centre of Excellence for Examination of Regulatory Role of Nitric Oxide in Civilization Diseases, Slovak Academy of Sciences, Bratislava, Slovakia; ^2^Institute of Physiology, Faculty of Medicine, Comenius University, Bratislava, Slovakia; ^3^Institute for Heart Research, Slovak Academy of Sciences, Bratislava, Slovakia; ^4^Institute of Medical Physics, Biophysics, Informatics and Telemedicine, Faculty of Medicine, Comenius University, Bratislava, Slovakia; ^5^Institute of Medical Chemistry, Biochemistry and Clinical Biochemistry, Faculty of Medicine, Comenius University, Bratislava, Slovakia

In the article titled “(−)-Epicatechin Prevents Blood Pressure Increase and Reduces Locomotor Hyperactivity in Young Spontaneously Hypertensive Rats” [1], there was an error in the section “2.1. Animals and Treatment,” where the sentence “Concentrated Epi solution (100 mg/mL) was prepared fresh every day before administration to rats by dilution of Epi in tap water (85°C, 3 min, in water bath)” should be corrected to “Concentrated Epi solution (10 mg/mL) was prepared fresh every day before administration to rats by dilution of Epi in tap water (85°C, 3 min, in water bath).”
