# Supplementary material for: (−)-Epicatechin Prevents Blood Pressure Increase and Reduces Locomotor Hyperactivity in Young Spontaneously Hypertensive Rats
Source: Oxid Med Cell Longev. 2016 Nov 3;2016:6949020. doi: 10.1155/2016/6949020 (PMC5112311; doi:10.1155/2016/6949020)
Supplement: Supplementary file 1 — Creatinine, uric acid and urea were determined in blood plasma at the end of treatment. All parameters were measured in the accredited medical laboratory Synlab Slovakia Ltd. (Bratislava, Slovakia). Results are expressed as mean ± SEM and they were analyzed by Student's T-test. [file 6949020.f1.docx]

**Supplementary information**

The nonprotein nitrogen fraction (NPN) in blood consists of about 15 compounds of clinical interest. Urea, uric acid, creatine and creatinine are four major NPN components and are routinely determined in clinical settings to monitor renal function (1).

As the relatively high dose of (-)-epicatechin (Epi) ~100 mg/kg/day was used in this study, we determined these markers to reveal whether the given dose of Epi is safe or if it produces adverse side effects to kidneys.

Creatinine, uric acid and urea were determined in blood plasma at the end of treatment. All parameters were measured in the accredited medical laboratory Synlab Slovakia Ltd. (Bratislava, Slovakia). Results are expressed as mean ± SEM and they were analyzed by Student's T-test.

No significant differences were observed between the control (n = 8) and Epi-treated rats (n = 10) as it is shown in the table. Thus, these results showed that two weeks of oral Epi treatment had no negative effects on renal function in spontaneously hypertensive rats.

|  | **Controls** | **Epi** |
| --- | --- | --- |
| Creatinine (µmol/l) | 20.6±0.9 | 20.9±0.7 |
| Uric acid (µmol/l) | 37.0±2.3 | 45.1±3.7 |
| Urea (mmol/l) | 6.7±0.2 | 7.0±0.2 |

Reference

1. Gowda S, Desai PB, Kulkarni SS, Hull VV, Math AA, Vernekar SN. Markers of renal function tests. N Am J Med Sci. 2010 Apr;2(4):170-3.
